# Feasibility and impact on daytime sleepiness of an experimental protocol inducing variable sleep duration in adolescents

**DOI:** 10.1371/journal.pone.0218894

**Published:** 2019-06-21

**Authors:** Tori R. Van Dyk, Nanhua Zhang, Angela Combs, Taylor Howarth, Catharine Whitacre, Shealan McAlister, Dean W. Beebe

**Affiliations:** 1 Department of Psychology, Loma Linda University, Loma Linda, California, United States of America; 2 Cincinnati Children’s Hospital Medical Center, Cincinnati, Ohio, United States of America; 3 Department of Pediatrics, University of Cincinnati College of Medicine, Cincinnati, Ohio, United States of America; Goteborgs Universitet, SWEDEN

## Abstract

Although most research on sleep and adolescent health has focused on how long each youth sleeps on average, variability in sleep duration may be just as problematic. Existing findings have been inconsistent and unable to address cause-effect relationships. This study piloted an experimental protocol to induce sleep variability and explore its impact on daytime sleepiness in adolescents. Healthy adolescents aged 14–17 participated in a 3-week, at-home protocol. Sleep was monitored by sleep diaries and actigraphy. Following a run-in period to stabilize wake times (set at 6:30am throughout the protocol), participants were randomly counterbalanced across two 5-night experimental conditions. Bedtimes were consistent at 11:00pm during the stable sleep condition (7.5-hour sleep period each night) but changed on alternating nights during the variable sleep condition (ranging from 9:30pm to 12:30am) so that sleep duration averaged 7.5 hours across the condition with a standard deviation of 1.37 hours. Difficulty waking was assessed each morning and daytime sleepiness was assessed by end-of-condition parent- and adolescent-reports. Of the 20 participants who completed the study, 16 met the predetermined adherence definition. For those who were adherent, there were no differences in overall sleep duration between the stable and variable sleep conditions (*p*>.05) but adolescents had 58.6 minutes greater night-to-night variation in sleep duration in the variable condition (*p* < .001). Across all nights, youth reported greater difficulty waking following nights of shorter assigned sleep (*p* = .004) and greater overall sleepiness during the variable condition (*p* = .03). It is feasible to experimentally vary how long adolescents sleep on a nightly basis while holding average sleep duration constant. Such a protocol will promote tests of the acute effects of day-to-day changes in sleep duration on health.

## Introduction

Adolescents do not obtain a sufficient amount of sleep on most nights [1]. The impact of inadequate sleep on cognitive, emotional, and physical health has been demonstrated in observational, longitudinal, and experimental studies [2–5], making the chronically short sleep of adolescents a public health crisis. However, beyond each adolescent’s average sleep duration, night-to-night variability in sleep duration and timing is emerging as a possible impediment to healthy functioning [6, 7]. Adolescents may be at particular risk for variable sleep night-to-night given rapid change in sleep physiology, increases in independence around bedtimes, and added and often varying academic, extracurricular, and environmental demands [7]. In fact, increased variability in sleep is associated with increasing age and pubertal development in youth, making adolescence a period marked not only by shortened sleep, but also unstable sleep patterns [6].

A recent systematic review found that greater sleep variability correlates with poor behavioral functioning, suboptimal sleep environments and schedules, and some aspects of physical health [6]. Indeed, some have suggested that variable sleep may have an even greater impact on adolescent wellbeing than overall sleep duration [7, 8]. However, there have been striking inconsistencies in findings, which may have been due to differences in research designs, measures, and statistical analyses [6]. Many studies fail to control for average sleep duration in analyses, which prevents the unique examination of the contribution of sleep variability in predicting functioning. On a more fundamental level, correlational data cannot establish the presence and direction of causation. For example, even though some studies have shown a correlation between inconsistent sleep and behavioral disturbance, it is not clear which causes the other or whether a third variable (e.g., genetics, chaotic family environment) might cause both [6]. As a result, while the common clinical recommendation to maintain a consistent sleep schedule (e.g., [9]) may have anecdotal support, its empirical foundation remains unclear.

Large-sample experimental studies that manipulate sleep variability/stability are needed, but before the field can undertake such work, it is of fundamental importance to first demonstrate that such studies are even feasible. Adolescence is a notorious period of non-adherence to medical directives [10], so having adolescents stabilize or increase variability in their sleep within the context of an adult-led experimental protocol is far from certain. Developing and demonstrating the feasibility of such an experimental protocol would open the door to more definitive tests of the health consequences of variable sleep during adolescence, in service of developing empirically-supported clinical and public health recommendations.

We have addressed this fundamental first step by developing an experimental manipulation of night-to-night variability in sleep duration, in which a more consistent sleep schedule is compared to a more variable one, while holding the overall average sleep duration constant. Because no such protocol has been attempted with adolescents, and because adolescent adherence to any such protocol is unknown, the primary aim of this paper is to describe the protocol and present pilot data on feasibility and adherence. Secondarily, we explored the impact of the protocol on daytime sleepiness.

## Methods

This study was approved by the Cincinnati Children’s Hospital Institutional Review Board (approval #2016–2307). Written parental consent and adolescent participant assent were obtained (see details below).

### Participants

Healthy adolescents aged 14–17 were recruited from community flyers, emails sent within a large regional children’s hospital, and from a participant waitlist for a separate study examining the effect of changes in sleep duration on symptom reporting in healthy adolescents (c.f., [11]). Exclusion criteria included parent- or adolescent-report of a psychiatric or neurological disorder; intellectual disability; adolescent refusal to refrain from high-vigilance tasks; use of medication impacting sleep or daytime alertness; symptoms of obstructive sleep apnea or restless leg syndrome; highly atypical sleep (<6 hours or >10 hours of self-reported school-night total sleep time); inflexible obligations requiring bedtime later than 9:30pm or waking prior to 6:30am; daily consumption of >1 coffee or “energy drink” or >2 caffeinated sodas; or body mass index >30. Although we excluded on the basis of atypically short sleep, we did not explicitly assess or exclude for insomnia.

### Procedures

Study procedures were approved by the local Institutional Review Board and were conducted during summer break to avoid unintended adverse effects on school performance. Telephone screenings were conducted with interested families to assess eligibility and verbally consent participants. At the initial office visit, eligibility was confirmed and written parental consent and adolescent assent were obtained. Prior to the initial visit, actigraph wristwatches with run-in period instructions were sent to families via mail and discussed via telephone.

**Fig 1:**
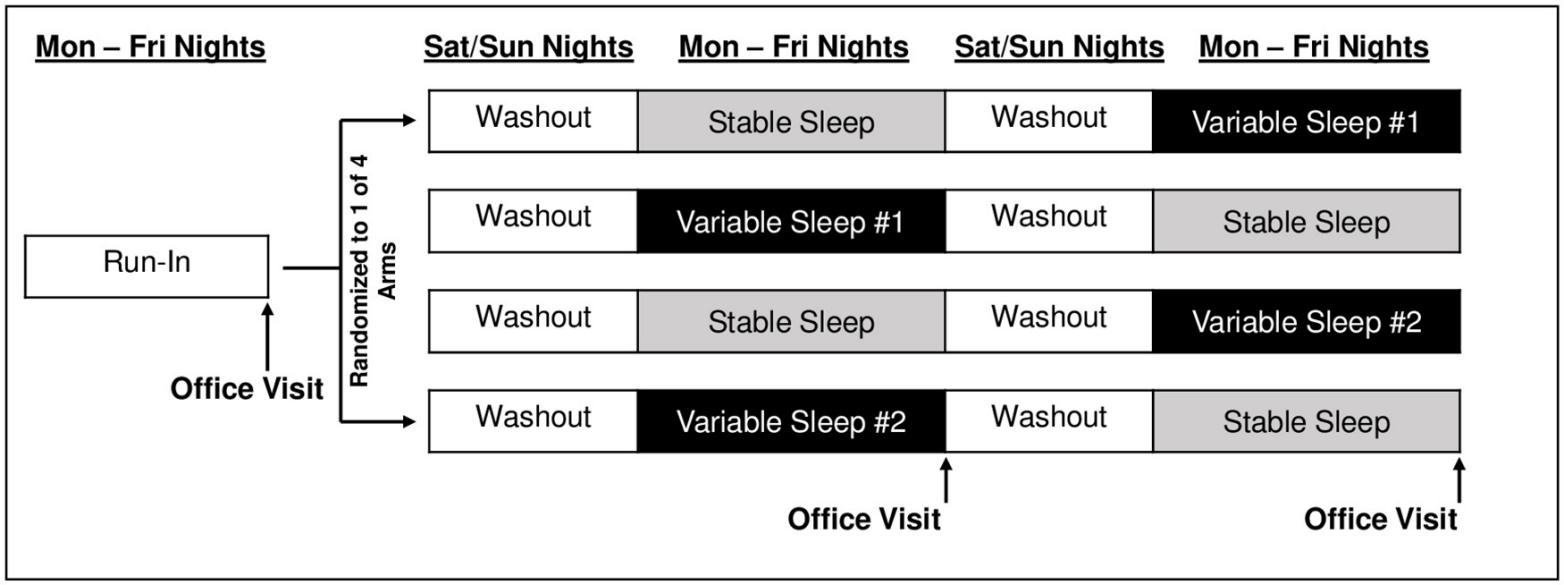
Depiction of the 3-week sleep manipulation protocol.

See Fig 1 for a depiction of the 3-week protocol and Table 1 for details regarding prescribed bedtimes and sleep durations for each day of the study. To maximize ecological validity and generalizability to non-laboratory settings, adolescents slept at home with sleep monitored by nightly sleep diaries and actigraphy. Each condition was comprised of five (Monday-Friday) nights, and each family attended an office visit on the Saturday morning following each condition. At office visits, data from sleep monitors were reviewed and instructions for the next experimental condition were provided with both the parent and adolescent present. Parents and adolescents also independently completed outcome measures at this visit.

**Table 1:** Assigned bedtimes and sleep period lengths for each night within each sleep manipulation condition.

Condition *(Average duration and variability)*	Mon	Tue	Wed	Thur	Fri	Sat	Sun
Run-In Period	Self-Selected						
Stable Sleep Condition *(7*.*5 hrs duration; no variability)*	11:00pm 7.5 hrs.						
Variable Sleep Condition #1 *(7*.*5 hrs duration; 1*.*37 hrs variability*)	10:00pm 8.5 hrs.	12:30am 6 hrs.	10:00pm 8.5 hrs.	12:30am 6 hrs.	10:00pm 8.5 hrs.		
Variable Sleep Condition #2 *(7*.*5 hrs duration; 1*.*37 hrs variability)*	12:00am 6.5 hrs.	9:30pm 9 hrs.	12:00am 6.5 hrs.	9:30pm 9 hrs.	12:00am 6.5 hrs.		
Washout Weekend *(8*.*5 hrs duration; no variability)*						10:00pm 8.5 hrs.	

Note: Wake times were held constant at 6:30am throughout the protocol.

The run-in period occurred prior to the first office visit and was comprised of five nights during which the adolescent was instructed to wake at 6:30am each morning but could self-select their bedtime. This “run-in” period was not intended to be a baseline assessment of typical sleep. Rather, because sleep schedules can “drift” during non-school times, the run-in period was intended to stabilize the participants’ circadian rhythm around a common school-year rise-time and to screen for non-adherence before the experimental weeks.

Participants were then randomized to one of four experimental arms. Within each arm, participants experienced the Stable Sleep Condition (STB) and one of two Sleep Variability Conditions (VAR) with participants randomly assigned to the order in which the experimental conditions were experienced. To promote similar states of rest before STB and VAR, each was preceded by a 2-night “washout weekend” (Saturday and Sunday night), in which bedtimes and wake times were set to allow 8.5 hours in bed with no induced variability. Because this was the first study of experimentally manipulated sleep variability (so appropriate washout period was unknown), the length of washout was guided by our prior experimental studies of sleep duration in adolescents, which found no order effects (i.e., sufficient washout) on a mood, behavior, and dietary outcomes using a similar 2-night washout [11–13]. The prescribed sleep period (bedtime to wake time) across the STB and both VAR conditions averaged 7.5 hours and the wake time was held constant at 6:30am. The goal was to create sleep durations and rise times that were, on average, similar to norms for US adolescents on school days [1].

As shown in Table 1, bedtimes varied depending on the condition to either stabilize sleep (i.e., 7.5 hours prescribed each night) or induce variability so that the nightly sleep period changed while maintaining a weekly average of 7.5 hours. During VAR, bedtimes were set so that the sleep period across nights had a standard deviation of 1.37 hours. This degree of variability was chosen to slightly overshoot the standard deviation of 1.2 hours that has been observed in “naturally occurring” adolescent sleep [8]. Since the manipulation had an odd number of nights (5 nights, representing a typical school week), two versions of the VAR condition were designed so that half of participants alternated three nights of shorter sleep with two longer and half got two nights of shorter sleep with three longer. This allowed us to explore whether there were differences in adherence or feasibility between these options. Bedtimes across the two versions of VAR were designed to average 11:00pm when pooled across all five days of the condition.

To support adherence to the protocol, at the study visit prior to starting each experimental condition, instructions for the following week’s sleep schedule were discussed with the adolescent and parent present. Potential challenges to keeping the schedule were identified and problem-solved and written, night-by-night instructions were provided. Also, daily text messages were sent each evening to remind participants of their bedtime for that night.

Participants were compensated $50 for attending each of the first and second study sessions and $75 for the third session. They also received $25 during the STB and VAR conditions if they pressed the actigraph event marker (to indicate when they went to bed) within 15 minutes of the instructed bedtime at least 4 out of 5 nights (see additional information regarding ensuring compliance to the protocol under Sleep Monitoring below). Participants could also earn up to $30 during the second and third week for completing an outcome unrelated to the present study.

### Measures

#### Demographics

Family income and adolescent sex, age, race/ethnicity, and height and weight (both objectively measured by researchers) were obtained during the first office visit.

#### Sleep monitoring

Adolescents completed nightly sleep diaries to accompany objective actigraphy (SleepWatch; Ambulatory Monitoring Incorporated, Ardsley, NJ). Sleep data from actigraphy and diaries were reviewed by staff at the end of each condition with the adolescent and parent present. Any discrepancies between the sleep diary, actigraph event marker, and actigraphy movement data were resolved during this session to best determine bedtimes and wake times. Potential artifacts (e.g., removal of watch, wearing watch too loosely, watch malfunction) were identified and bedtimes and wake times were confirmed. When artifacts were identified that could affect our measured sleep outcome variables, data for this night was removed so as to not distort results. This was very rare; there were no artifacts removed during VAR and only two nights (across two different participants) removed during STB. Once screened for artifacts, actigraphy has been shown to have greater than 90% concordance with EEG-defined sleep parameters in healthy adolescents [14]. Actigraphy-derived sleep onset, offset, duration (offset minus onset), and efficiency (percent of the period between sleep onset and offset spent in sleep) were determined for each night using the validated Sadeh algorithm. Because it can be difficult to verify actual bedtime in this age range in the home environment, we did not analyze sleep onset latency. Sleep variability for each participant and condition was calculated as the standard deviation of sleep duration across the 5 nights of that condition.

#### Difficulty waking

The sleep diary included a daily report of how hard it was to wake up each morning. Adolescents responded on a 5-point scale (very easy– 1 to very difficult– 5). Similar daily sleep diary questions regarding difficulty waking have been used in experimental sleep research and have been shown to be sensitive to overall changes in sleep duration across a 5-night period [12].

#### Daytime sleepiness

Overall sleepiness was assessed at the end of each condition by both parents and adolescents using a 5-item questionnaire previously adapted from the Child Sleep Habits Questionnaire [15] (see [12] for details). Adolescents and parents responded to questions on a 4-point scale with higher scores indicating greater daytime sleepiness. This scale has been previously validated and found to be sensitive to the effects of 5-night manipulations of adolescents’ sleep duration [12, 13, 16].

### Analysis plan

The primary aim of the study was to assess protocol feasibility, participant retention (i.e., attendance to all three study visits), and adherence (defined *a priori* for each participant as ≥30 minutes variability in sleep duration during VAR compared to STB and ≤30 minutes difference in overall sleep duration between conditions). Intent-to-treat analyses using the full sample (n = 20) and analyses using the adherent subsample (n = 16) were conducted to assess for protocol feasibility and impact on sleepiness. Because there were very few differences between these sets of findings, we present detailed results only for the adherent subsample, highlighting in the text the few minor discrepancies compared to full-sample findings. Actigraph parameters were compared across conditions (i.e., VAR vs. STB), study arms, and at the daily-level by assigned sleep period duration using linear mixed effect models which accounted for the dependence of measurements from the same subjects. The order in which participants experienced conditions was also analyzed as a potential moderator and post-hoc analyses to compare the conditions (VAR vs. STB) within the order were conducted when there was significant order moderation. Finally, we explored whether demographic moderators affected results; they did not and are not included in models presented.

A secondary aim of the study was to assess the impact of variable sleep on daytime sleepiness, measured at the daily-level with self-reports of difficulty waking and across conditions with both self- and parent-reports of daytime sleepiness. Linear mixed effect models were used for modeling daytime sleepiness. Generalized linear mixed effect models with cumulative logit link were used to model self-reported difficulty waking.

## Results

### Participant characteristics, flow, and retention

This pilot study was designed to enroll participants until 20 completed all three weeks. Twenty-six adolescent-parent dyads were recruited and scheduled to participate. Three of these withdrew during the run-in period and 2 were dropped at the initial visit due to nonadherence to the run-in sleep instructions (i.e., excessive sleeping in). The remaining 21 participants were randomized, but one participant (assigned to STB first) was dropped at the second office visit due to nonadherence (i.e., staying up much later than assigned bedtime). Thus, 20 participants were retained and attended all three study visits. Of these, average variability in sleep duration during VAR ranged from 52.39 to 136.42 minutes, with all but three participants demonstrating an average variability above 60 minutes. Sixteen of the twenty participants (80%) completing the study met the predetermined adherence definition of demonstrating ≥30 minutes variability in sleep duration during VAR compared to STB with ≤30 minutes difference in overall sleep duration between conditions. See Table 2 for demographic information for the 23 participants attending the first office visit, the 20 who completed all three visits, and the 16 who were deemed adherent to the protocol. As can be seen, although the sample was small, it was relatively diverse and there was little evidence of systematic attrition by demographics.

**Table 2:** Demographic information for participants attending at least the initial office visit (“full sample”), those attending all three office visits (“completers”), and those adherent to the study protocol (“adherent”).

Demographics	Full Sample	Completers	Adherent
N	23	20	16
Age	14.87 ± 1.01	14.90 ± 1.07	15.13 ± 1.09
Female Gender	12 (52.2%)	12 (60.0%)	10 (62.5%)
Race^a^			
*White*	14 (63.6%)	13 (68.4%)	10 (62.5%)
*Black*	5 (22.7%)	4 (21.1%)	4 (25.0%)
*Biracial*	3 (13.6%)	2 (10.5%)	2 (12.5%)
Income^a^			
*under $49*,*000*	5 (22.7%)	5 (26.3%)	5 (33.3%)
*$50*,*000-$79*,*000*	4 (18.2%)	3 (15.8%)	3 (20.0%)
*$80*,*000-$99*,*000*	5 (22.7%)	4 (21.1%)	2 (13.3%)
*$100*,*000-$124*,*000*	4 (18.2%)	4 (21.1%)	2 (13.3%)
*$125*,*000 or more*	4 (18.2%)	3 (15.8%)	3 (20.0%)

^a^One participant declined to provide full demographic information.

### Protocol feasibility

**Fig 2:**
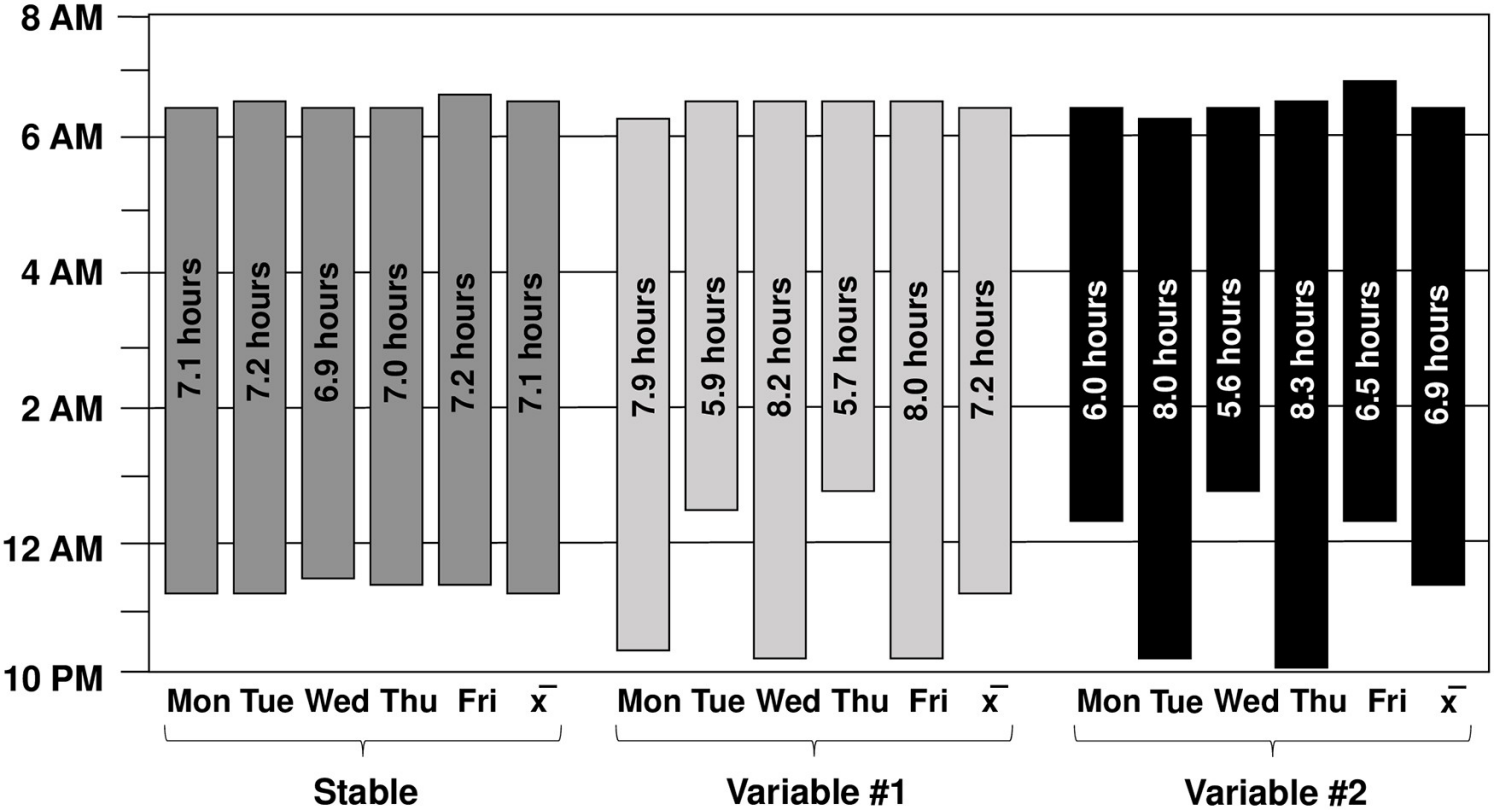
Actigraphy-derived daily-level sleep patterns for the stable sleep condition and each variable sleep condition. Mean sleep onset is represented by the bottom of each bar and wake time by the top with average sleep duration printed within each bar. Depiction of relative stability and variability across experimental conditions in regards to daily-level sleep onset, offset, and duration for adolescents completing the protocol with adequate adherence.

Table 3 summarizes daily-level actigraph parameters across the run-in and both experimental conditions and Fig 2 depicts daily-level sleep onset, offset, and duration across experimental conditions. Across all washout nights, average sleep duration was 7.72 ± .40 hours. Consistent with our goal of developing overall equivalent versions of the variable sleep conditions, when pooled across each 5-night span there were no average differences between the variable sleep conditions (Variable #1 and Variable #2) in regards to overall sleep duration, variability, onset time, offset time, and sleep efficiency (all *p*>.05). Therefore, our subsequent analyses pooled the two variability conditions and focused on the differences between VAR and STB. As expected, average sleep duration did not differ between STB and VAR, *F*(1, 141) = .11, *p* = .74. None of these findings differed in intent-to-treat analyses. Overall, there were no cross-condition differences in *actual* sleep duration compared to *assigned* sleep duration for the adherent subsample, *F*(4, 138) = 1.81, *p* = .13. Intent-to-treat analyses in the full sample reflected the impact of those who were nonadherent to the study protocol, such that there were modest differences in actual compared to assigned durations (*p* = .04).

**Table 3:** Daily-level actigraph parameters across the stable sleep condition and each variable sleep condition for adherent participants.

Protocol Condition	Day of Week					Condition Averages	
	Mon	Tue	Wed	Thur	Fri	Duration	Variability
**Run-In (n**^a^**)**						400.27 ± 52.27	45.80 ± 17.33
*Duration (min)*	389.67 ± 61.92	381.27 ± 62.65	412.38 ± 59.84	414.75 ± 68.68	398.38 ± 85.44		
*Onset Time*	12:01 ± 1:03	12:07 ± 1:06	11:47 ± :57	11:36 ± 1:08	12:17 ± 1:28		
*Offset Time*	6:31 ± :15	6:28 ± :19	6:39 ± :23	6:30 ± :25	6:54 ± :55		
*Efficiency*^b^ *(%)*	84.18 ±10.84	89.06 ± 7.07	88.03 ± 12.40	90.91 ± 6.69	90.23 ± 6.93		
**Stable (n = 16)**						424.63 ± 21.32	22.53 ± 11.47
*Duration (min)*	425.25 ± 19.89	429.56 ± 33.06	413.56 ± 40.97	420.81 ± 22.63	433.29 ± 33.62		
*Onset Time*	11:21 ± :15	11:20 ± :29	11:32 ± :37	11:26 ± :22	11:27 ± :17		
*Offset Time*	6:26 ± :09	6:30 ± :13	6:25 ± :10	6:27 ± :07	6:40 ± :31		
*Efficiency*^b^ *(%)*	87.47 ± 10.98	88.09 ± 8.17	88.71 ± 9.39	89.71 ± 9.85	87.90 ± 12.84		
**Variable #1 (n = 7)**						431.49 ± 15.08	77.08 ± 9.72
*Duration (min)*	476.14 ± 16.31	356.29 ± 19.86	493.43 ± 39.03	342.0 ± 22.28	482.57 ± 11.0		
*Onset Time*	10:23 ± :16	12:32 ± :22	10:17 ± :12	12:50 ± :26	10:19 ± :12		
*Offset Time*	6:19 ± :16	6:28 ± :05	6:30 ± :33	6:32 ± :11	6:28 ± :04		
*Efficiency*^b^ *(%)*	91.11 ± 5.86	92.25 ± 4.39	90.40 ± 6.30	91.40 ± 5.62	92.37 ± 5.73		
**Variable #2 (n = 9)**						413.22 ± 26.93	84.21 ± 23.80
*Duration (min)*	361.78 ± 34.89	480.78 ± 47.91	333.0 ± 75.54	499.56 ± 35.56	391.0 ± 60.0		
*Onset Time*	12:20 ± :20	10:17 ± :37	12:52 ± 1:13	10:08 ± :33	12:17 ± :34		
*Offset Time*	6:22 ± :20	6:17 ± :22	6:24 ± :09	6:27 ± :06	6:48 ± 1:00		
*Efficiency*^b^ *(%)*	89.65 ± 8.84	89.51 ± 10.0	88.19 ± 13.77	89.15 ± 11.04	87.77 ± 11.13		

^a^Due to missing data during the run-in period, n = 15 on Monday and Tuesday and n = 16 on Wednesday through Friday of the run-in.

^b^Efficiency represents the percent of the period between sleep onset and offset spent in sleep.

Consistent with study aims, adolescents demonstrated 58.6 minutes greater variability in sleep duration during VAR compared to STB, *F*(1, 15) = 181.70, *p* < .001. The order in which experimental conditions were experienced moderated this effect. Although there were significant differences in sleep variability regardless of which condition was experienced first, those assigned VAR first had even greater differences between conditions than those assigned STB first (67.4 vs. 47.2 minute difference, respectively). Findings were unchanged in intent-to-treat analyses.

There were no differences in sleep efficiency between study conditions, *F*(1, 141) = 3.65, *p*>.05, for the adherent sample. In intent-to-treat models with the full sample, there were differences in sleep efficiency between conditions (*p* = .02) with adolescents averaging 1.6% less efficient sleep during STB compared to VAR. However, this difference likely has negligible clinical significance.

Table 4 summarizes actigraphy-derived sleep parameters across the five different assigned sleep periods (450 minutes for STB nights, 360–540 minutes during VAR). As instructed, adolescents woke in the morning near 6:30am with no difference in wake time across the assigned sleep periods, *F*(1, 141) = .39, *p* = .54. There were differences in sleep onset time, *F*(4, 138) = 120.90, *p* < .001. As expected, actual sleep duration increased monotonically with longer assigned sleep; while this was most evident across the three “middle” assigned sleep periods, those sleep periods were also spaced further apart than those at either end. Although more subtle, the latency between assigned bedtime and actual sleep onset increased with longer assigned sleep periods, from 16 minutes on average when assigned a 360 minute (6 hour) sleep period to 38 minutes on average when assigned a 540 minute (9 hour) sleep period. There were no differences in sleep efficiency by assigned sleep period, *F*(4, 138) = 1.08, *p* = .37 (data not shown). Intent-to-treat analyses yielded the same pattern of results.

**Table 4:** Actual sleep onset, offset, and duration (as measured by actigraphy) across varying assigned sleep periods and bedtimes for adherent participants.

Actigraph Parameter	Assigned Sleep Period (in minutes) and Bedtime					Cross-Period Comparisons
	360 min	390 min	450 min	510 min	540 min	
	12:30am	12:00am	11:00pm	10:00pm	9:30pm	
*Onset Time*	12:46am	12:25am	11:25pm	10:25pm	10:08pm	360 = 390<450<510 = 540
*Offset Time*	6:31am	6:31am	6:29am	6:27am	6:21am	Not significant
*Duration (min)*	344.5	365.6	424.5	481.7	493.8	360 = 390<450<510 = 540

### Impact on sleepiness

At the daily-level there were significant differences in adolescent-reports of difficulty waking based on assigned sleep period, *F*(4, 134) = 4.10, *p* = .004, with the odds of having difficulty waking being significantly greater on nights with an assigned sleep duration of 360 minutes compared to 510 minutes (OR = 6.80, 95% C.I.: 2.00–23.11) and 540 minutes (OR = 9.74, 95% C. I.: 2.49–38.18). These odds ratios, which are comparable to Cohen’s *d* values exceeding 1.0, correspond to large effect sizes [17]. Not only did youth report more difficulty waking following shorter nights of sleep, but overall, adolescents reported more daytime sleepiness following VAR (*M* = 10.25, *SE* = 0.52) compared to STB (*M* = 9.31, *SE* = 0.52), *F*(1, 15) = 5.42, *p* = .03, *d* = 0.52 (medium to large effect size). Parents did not report significant end-of-condition differences in their child’s daytime sleepiness, *F*(1, 15) = .46, *p* = .51, *d* = 0.15. Findings were unchanged in intent-to-treat analyses.

## Discussion

This study demonstrates that it is feasible to conduct a protocol which induces variable sleep duration while holding overall sleep duration constant, allowing for a direct comparison between variable and stable sleep duration. Of 23 participants attending the baseline visit, approximately 30% were lost to being ineligible or non-adherent over the course of the study. However, of 21 participants who met eligibility for randomization, 20 (95%) completed both the sleep stabilization and variability induction conditions. Four were then unable to meet *a priori* criteria for adherence to sleep period instructions, but findings were nearly identical across full-sample intent-to-treat analyses and analyses that focused only on the 16 deemed adherent. Overall, data point to the protocol being feasible in the large majority of our healthy adolescent participants.

Both experimental conditions yielded a 5-night average of ~7 hours of nightly sleep; not only does the cross-condition equivalence eliminate the potential for confounding between sleep duration average and sleep variability, it is also realistic, approximating the average school night sleep duration for US adolescents [1]. The manipulation “dose” was also realistic: the variable sleep condition yielded night-to-night variation ~80 minutes; similar to that found in observational studies of adolescents [8]. In contrast, night-to-night variability during the stable sleep condition was around 23 minutes, a highly significant drop in our within-subjects analyses and over 1-1/3 standard deviations below population norms [8]. Further, the sleep-at-home nature of the protocol promotes the generalizability of findings, reduces costs associated with on-site or inpatient studies, and facilitates recruitment and retention [12].

Although our primary goal was to test feasibility of this new protocol, we explored its effect on sleepiness to illustrate one potential application. Experimentally manipulating overall sleep duration across several nights affects sleepiness and fatigue [13, 16], but the current protocol allowed for a novel test of the impact of *variable* sleep duration on daytime sleepiness. Results suggest that not only do youth report greater difficulty waking up on mornings following short sleep, they also report more overall daytime sleepiness after experiencing five days of variable sleep compared to stable sleep. This is particularly noteworthy because the stable sleep schedule centered closely around 7 hours of sleep, chronically less than the recommended 8–10 hours [18], whereas the variable sleep condition allowed for 8 or more hours of sleep some nights. Our findings suggest that sporadically getting 8 or more hours of sleep interspersed with much shorter nights may overall feel *worse* to adolescents than getting consistent but modestly shortened sleep. However, our sleepiness findings are preliminary and, while parent-report effects were in the same direction, they were statistically non-significant. Future research is needed to understand this discrepancy and to examine additional outcomes in larger samples. Even so, our preliminary findings (a) point to one way adolescents may benefit from keeping more regular sleep schedules, (b) support correlational studies finding sleep stability to be important to healthy functioning [7, 8], and (c) illustrate the potential applications of this novel experimental protocol.

Clinically, recommendations to promote consistent sleep-wake schedules in youth are longstanding and based on stabilizing the circadian rhythm and improving sleep quality [19]. The development of this protocol allows for the possibility to experimentally evaluate established claims regarding the benefit of promoting stability. Preliminary findings from the study support stabilizing sleep as it relates to sleepiness and reinforces the idea that practitioners should not just assess for average sleep duration but should evaluate sleep at the daily-level with the use of sleep diaries and/or actigraphy. Further, providers should intervene around sleep stability when indicated by identifying barriers to consistent sleep-wake schedules, evoking internal motivation to stabilize sleep (e.g., improvement in daytime sleepiness), and involving caregivers in encouraging set bedtimes and wake times.

### Limitations and conclusions

This was a pilot study with a small sample of youth. Although promising, findings from this proof-of-concept study should be replicated in larger and more diverse samples. For the initial test of this protocol, we did not attempt a random sampling approach. The small sample prevents definitive conclusions regarding feasibility across demographic groups. Future researchers should aim to recruit more diverse, representative samples while also considering the potential challenges of retaining subgroups. Although this small sample was clearly sufficiently powered to detect our primary within-subjects outcome measure of variability in sleep duration, it lacked power to detect smaller effects or those that occur between (not within) subjects; this may have contributed to null findings on parent-reports of daytime sleepiness or demographic moderators. Also, the focus was on protocol development, description, and feasibility, with only a preliminary test of effect on a single outcome domain: subjective sleepiness. Although previously validated and used in prior experimental sleep research, the psychometric properties of our measures of sleepiness are lacking. There is more work to be done even using other sleepiness measures, let alone the many other outcomes that have been investigated vis-à-vis sleep duration in experimental work or sleep variability in correlational studies [6]. Some outcomes may not lend themselves to this short-term protocol, though important proxy variables might be possible (e.g., assessing short-term changes to diet or physical activity rather than longer-term body mass effects). Considering the protocol maintained a consistent 6:30am wake time, it is possible that effects could have differed across chronotypes. We did not assess for chronotype in our sample but future protocols may wish to include this variable. The consistent 6:30am wake time applied not only to the experimental conditions but also to the run-in period. Although this period was necessary to help stabilize circadian rhythms before the experimental conditions, it also may have altered the adolescents’ typical night-to-night variability and/or overall sleep duration. Because it was not a true baseline, we were unable to assess if the protocol differentially affects adolescents who typically have greater or less sleep duration or variability. Finally, our study was conducted during the summer months to avoid ethical concerns around inducing short or more variable sleep during the school year. It is possible that the impact of variable sleep would be different during the school year when the environment is different (e.g., cooler temperatures) and adolescents’ eating, sleeping, activity, and social patterns tend to be altered. Future researchers might desire to design a school-year protocol (e.g., [16]), incorporate assessments of environmental contributors to sleep variability (e.g., temperature, noise), add a true baseline, lengthen the protocol, consider shifts in sleep phase, lengthen the between-condition wash-out conditions (particularly for constructs that researchers are concerns will be slow to normalize), intensify the degree of induced variability, test different mean sleep durations, or alter the pattern of short-vs-long sleep periods.

In designing future work, logistical and economic factors are important. The ability to recruit healthy adolescents will vary across communities. We provided financial compensation to participants for attending office visits and adhering to the sleep protocol; a fully adherent participant earned $225. Actigraphs were the only device used. Costs and basic functionality of actigraphs have been reducing dramatically as technology improves and marketplace competition increases. At the time this paper was written, well-validated actigraphs with basic functionality could be obtained for less than $300 each and could be re-used many dozens of times. Finally, staffing needs would vary depending on the number of participants attending each office visit at one time. At a minimum, for individual participant appointments, one staff member trained in scoring actigraphy data would be required, in addition to staffing needed for recruitment, screening, scheduling, data management, and analyses.

This study lays important groundwork for such research. Although no sleep manipulation works for all participants or outcomes, the current protocol appears feasible even in the high-risk developmental stage of adolescence, and balances real-world applicability with a rigorous experimental, within-person, cross-over design which promotes well-powered statistical comparisons and causal conclusions.

## Supporting information

S1 Dataset Dataset including data underlying the present findings. (SAV)
